# Frankly, we do give a damn: improving patient outcomes with swearing

**DOI:** 10.1186/s40945-022-00131-8

**Published:** 2022-03-17

**Authors:** Nicholas B. Washmuth, Richard Stephens

**Affiliations:** 1 Department of Physical Therapy, Samford University, 800 Lakeshore Dr, Birmingham, AL 35229 United States; 2 School of Psychology, Keele University, Keele, Staffordshire ST5 5BG UK

**Keywords:** Swearing, Power, Force, Pain, Biopsychosocial

## Abstract

**Background:**

Words can change the way a patient thinks, feels, and performs. Swearing, or uttering a word that is considered taboo, is an often-ignored part of our language, even though over 50% of the population swears “sometimes” or “often”. If used correctly, within a biopsychosocial approach to care, swearing has the potential to significantly improve patient outcomes.

**Discussion:**

Swearing can create tighter human bonds and enhance the therapeutic alliance between a patient and a physical therapist. Improvements in social pain, physical pain tolerance, and physical pain threshold can occur by strategic swearing by our patients. Even physical performance measures, such as power and force could be enhanced if patients swear.

**Conclusion:**

Although the mechanism by which swearing is effective is unclear, swearing deserves attention in the physical therapy setting based on evidence indicating potential positive effects on patient outcomes.

## Introduction

We were first introduced to the quote, “words are, of course, the most powerful drug used by mankind”, in a previous Viewpoint, which discusses how the words used by physical therapists have the capacity to either heal or cause harm [1]. Like drugs, words have an ability to change the way another person thinks, feels, and performs. As physical therapists managing patients within a biopsychosocial approach, the language used may be just as important as, if not more important than, any examination finding or intervention.

Swearing, or uttering a word that is considered taboo, is an often-ignored part of our language due to the controversial nature of the topic and the potential negative consequences of swearing. In 1901, Patrick [2] concluded that swearwords are primarily used by soldiers, sailors, laborers, uneducated people, and criminals, and men make up the majority of these social groups. While swearing has been considered a predominately masculine activity, women now tend to swear as much, or even more often, then men [3]. Since swearing is considered taboo, these words are usually judged as shocking, and the swearer may be considered antisocial and offensive. Consequently, swearing can negatively impact how the swearer is perceived by others, which may lead to social isolation and depression. In groups of women with rheumatoid arthritis or breast cancer, Robbins et al. [4] found that swearing was related to increases in depressive symptoms, and this association remained significant even after accounting for variances in the depressive symptoms associated with disease severity. Interestingly, only swearing in the presence of others, and not swearing alone was correlated with increased depressive symptoms [4]. In specific contexts, swearing has been shown to produce negative outcomes.

Swearing may also be a sign of intelligence, is associated with less lying and deception at the individual level and higher integrity at the society level, and may be a sign of creativity [5]. The offensiveness and the positive or negative consequences of swearing is highly dependent on the context. While it is not fully understood why swear words are so powerful, it has been suggested that swearing is learned during childhood and that aversive classical conditioning contributes to the emotionally arousing aspect of swear words [6]. Swearers who disregard social codes and are at odds with the context, may offend the audience and break social convention, leading to negative consequences such as less positive attitudes towards the swearer [7]. This can occur by swearing in front of children or around people of higher status. To optimize the positive outcomes from swearing, physical therapists can use social codes cues of the situation which includes their intent, patient’s facial expressions, tone and gestures, and the relationship between the physical therapist and patient [7].

Humans have been swearing since the emergence of language [3] and is quite common, with evidence suggesting 58% of the population swears “sometimes” or “often” and less than 10% of the population report “never” or “rarely” swearing [8]. Most often, it is the swear word itself that is considered taboo rather than the semantic meaning it conveys. For example, talking about sexual intercourse by itself is not considered swearing; however, the *F-word* is a well-recognized swear word considered “very severe” by 71% of the population [9].

### Benefits of swearing

Swearing in the physical therapy setting should be used to accomplish specific goals, such as relief from pain or stress. When swearing is based on biopsychosocial utility, it may add significant value if used correctly. Swearing tends to be more tolerated in private settings and among peers as opposed to a more formal and public setting. Swearing can lead to tighter human bonds and create informal environments where people are more likely to be themselves [3]. Social groups depend on some degree of shared willingness to participate in risks or taboo practices, swearing being one of them. In the physical therapy setting, an improved relationship or positive connection between a patient and a physical therapist, otherwise known as the therapeutic alliance, has been linked to improvements in musculoskeletal pain.

Language used by the physical therapist and the patient can impact social, psychological, and biological factors, all of which heavily influence symptoms presentation and prognosis [1, 10]. Social pain, described as a feeling of suffering brought on when social connections are lost or threatened, is biologically coupled with physical pain [11]. Similarly, the therapeutic alliance that decreases physical pain also mitigates social pain [12]. Establishing a strong, positive therapeutic alliance is valuable for addressing the psychosocial influence of pain. Swearing has also been shown to reduce social pain [13], which may be related to improved social connections.

Swearing has also been found to decrease physical pain. Repeating a swear word while your hand is immerged in ice-water will allow you withstand the cold for 40 s longer, on average, compared to repeating a non-swear word [14]. This ice-water immersion yields scores for pain threshold (time at which pain is reported) and pain tolerance (time at which the hand is removed) and swearing has beneficial effects on both pain tolerance and threshold. It appears swearing is most effective at increasing pain thresholds among people who swear less often [14].

There is evidence that swearing out loud can also increase physical performance. Uttering a swear word every three seconds for the entire 30-s Wingate Anaerobic Power Test allows you to exert greater levels of peak power and average power compared to repeating a non-swear word [15]. Evidence also suggests you can exert a greater level of maximal force while squeezing a grip dynamometer while repeating a swear word [15]. In fact, many athletes admit to regularly using swearing words [16], which may be related to the improvements in physical performance that occur when swearing.

Swearing can modulate physical and social pain, and increase physical performance; however, we don’t yet know the mechanism by which swearing works. Stephens et al. [15] did not find measurable cardiovascular or autonomic arousal effects, with no clear changes in heart rate, skin conductance, or blood pressure when swearing. Therefore, increased muscular performance during swearing may be achieved by mechanisms other than sympathetic activation. Distraction of one’s attention away from a painful stimulus is known to reduce pain perception. It may be that we are distracting ourselves when we swear, thereby decreasing our pain perception. It is possible that swearing-induced distraction produced the improved performance during the Wingate Anaerobic Power Test and grip tasks, making it more tolerable to pedal hard and produce force while griping; however, future research is required to determine the mechanism by which swearing is effective.

What swear word should be used to get these pain and physical performance improvements? It is advised to use a swear word that you would use in response to banging your head accidentally [15]. If no clear swear words come to mind, the *S-word* and *F-word* are the two most common swear words [8, 9] and were used by many of the subjects in the research showing the positive effects of swearing. There is evidence that a patient needs to use an actual swear word, not a made up or bad sounding word, to elicit the pain and physical performance improvements. Stephens et al. [17] discovered that pain tolerance and pain threshold improved in subjects that repeated the *F-word*, but pain metrics did not improve when subjects repeated the made up swear words “fouch” or “twizpipe”.

To elicit the positive effects on pain and physical performance, without negative consequences, patients should not swear at the physical therapist. The research showing positive effects on pain and physical performance had their subjects swear out loud, not at a specific individual. Being the target of verbal aggression by asking a patient to swear at a clinician appears to lead to a high degree of distress among health care workers [18].

Due to the potential negative effects of swearing, physical therapists should carefully determine which patients are likely to experience the greatest benefit from swearing, without risking negative consequences. The patients most likely to benefit from swearing are those who have strong rapport and therapeutic alliance with their clinician [8], those who use swearing sparingly which will preserve the hypoalgesic effects of swearing [14], and those who can swear privately or among their peers [4].

## Conclusion

Patients’ dysfunctions develop from a combination of biological, psychological, and social factors that may also influence how their dysfunctions progress and their prognosis. Swearing deserves attention in the physical therapy setting based on evidence indicating positive effects on physical pain, social pain, and physical performance (Table 1). Swearing has positive and negative effects. Obviously, the possible use and effect of swearing may be highly dependent on its context. The relationship between the swearer and others in the social context, the formality of the situation, and the public or private nature of the situation are examples of such contextual factors that can influence the functionality of swearing. Many factors will play into whether including swearing will improve patient outcomes, one of which is the need for clinicians to have excellent relationship skills to help patients strategically incorporate swearing into their treatment plan. If words are the most powerful drug used by mankind, then the physical therapy profession should embrace swearing to change the way our patients think, feel, and perform.

**Table 1:** Possible Improvements in Patient Outcomes Due to Swearing

Improved Therapeutic Alliance	
Decreased Social Pain	
Increased Physical Pain Tolerance	
Increased Physical Pain Threshold	
Increased Physical Power	
Increased Maximal Force Development	

## Data Availability

Not applicable, no data.
